# Ultracontinuous Single Haplotype Genome Assemblies for the Domestic Cat (*Felis catus*) and Asian Leopard Cat (*Prionailurus bengalensis*)

**DOI:** 10.1093/jhered/esaa057

**Published:** 2020-12-11

**Authors:** Kevin R Bredemeyer, Andrew J Harris, Gang Li, Le Zhao, Nicole M Foley, Melody Roelke-Parker, Stephen J O’Brien, Leslie A Lyons, Wesley C Warren, William J Murphy

**Affiliations:** 1 Veterinary Integrative Biosciences, Texas A&M University, College Station, TX; 2 Interdisciplinary Program in Genetics, Texas A&M University, College Station, TX; 3 College of Life Sciences, Shaanxi Normal University, Xi’an, Shaanxi, China; 4 Frederick National Laboratory of Cancer Research, Leidos Biomedical Research, Inc., Frederick, MD; 5 Laboratory of Genomic Diversity-Center for Computer Technologies, ITMO University, Saint Petersburg, Russian Federation; 6 Guy Harvey Oceanographic Center, Nova Southeastern University, Fort Lauderdale, FL; 7 Department of Veterinary Medicine & Surgery, College of Veterinary Medicine, University of Missouri, Columbia, MO; 8 Bond Life Science Center, University of Missouri, Columbia, MO

**Keywords:** genome, Felidae, interspecies hybrid, trio-binning, PacBio

## Abstract

In addition to including one of the most popular companion animals, species from the cat family Felidae serve as a powerful system for genetic analysis of inherited and infectious disease, as well as for the study of phenotypic evolution and speciation. Previous diploid-based genome assemblies for the domestic cat have served as the primary reference for genomic studies within the cat family. However, these versions suffered from poor resolution of complex and highly repetitive regions, with substantial amounts of unplaced sequence that is polymorphic or copy number variable. We sequenced the genome of a female F1 Bengal hybrid cat, the offspring of a domestic cat (*Felis catus*) x Asian leopard cat (*Prionailurus bengalensis*) cross, with PacBio long sequence reads and used Illumina sequence reads from the parents to phase >99.9% of the reads into the 2 species’ haplotypes. *De novo* assembly of the phased reads produced highly continuous haploid genome assemblies for the domestic cat and Asian leopard cat, with contig N50 statistics exceeding 83 Mb for both genomes. Whole-genome alignments reveal the *Felis* and *Prionailurus* genomes are colinear, and the cytogenetic differences between the homologous F1 and E4 chromosomes represent a case of centromere repositioning in the absence of a chromosomal inversion. Both assemblies offer significant improvements over the previous domestic cat reference genome, with a 100% increase in contiguity and the capture of the vast majority of chromosome arms in 1 or 2 large contigs. We further demonstrated that comparably accurate F1 haplotype phasing can be achieved with members of the same species when one or both parents of the trio are not available. These novel genome resources will empower studies of feline precision medicine, adaptation, and speciation.

The cat family Felidae is a speciose and geographically dispersed mammalian radiation, containing many of the most charismatic and endangered apex predators on Earth. Decades of genetic analysis of the species from this lineage have been driven by veterinary medical interest in the domestic cat, and its use as a biomedical model (O’Brien et al. 1982; 2002). Furthermore, wild felid species have benefitted from the advances in and applications of domestic cat genome assemblies to studying their conservation biology and evolutionary history (e.g., Johnson et al. 2006; O’Brien et al. 2006; Luo et al. 2008; Abascal et al. 2016; O’Brien et al. 2017; Zhang et al. 2020). Several attributes of felid genomes are ideal for comparative genetic analysis, including the strong chromosomal collinearity between all species (Wurster-Hill and Centerwall 1982; Modi and O’Brien 1988; Davis et al. 2009), and the highest reported rates of meiotic recombination within mammals (Menotti-Raymond et al. 2003; Segura et al. 2013; Li et al. 2016a). There is also an extensive body of literature describing prolific interspecific hybridization within and between the major clades of the cat family, both in free-ranging populations and through human-controlled breeding (Gray 1972; Schwartz et al. 2004; Homyack et al. 2008; Trigo et al. 2008, 2013; Davis et al. 2015; Li et al. 2016b; Figueiró et al. 2017; Li et al. 2019). Previous studies have highlighted the role of interspecific hybrids in the generation of essential genomic tools. In particular, the Bengal cat cross (*Felis catus* x *Prionailurus bengalensis*) was instrumental in the development of the first feline genetic maps (Menotti-Raymond et al. 1999, 2003).

Although the process and accuracy of genome assembly has progressed substantially in the past decade towards the capture of the most repetitive sequences, assemblies of diploid genomes still suffer from several problems: the absence or collapse of long repetitive DNA, failure to resolve sites of high allelic variation between haplotypes, and the pseudohaploid representations of diploid genomes are artifactual representations of the original parental haplotypes. Trio binning was developed to sort and independently assemble divergent parental haplotypes from F1 hybrids using a combination of short-read Illumina and long-read PacBio sequences, and has been used to generate haploid assemblies for the parent species of several bovid interspecific and subspecific hybrids (Koren et al. 2018; Low et al. 2020; Rice et al. 2020). This approach exploits the high heterozygosity found in interspecies F1 hybrids that was originally used to develop comparative genetic maps that allowed divergent mammalian genomes to be aligned and compared (Lyons et al. 1997; Menotti-Raymond et al. 2003). These same attributes greatly simplify the phasing of parental haplotypes when applied to parent-offspring trios, notably those based on F1 interspecific hybrids. In addition to generating novel genomes from closely related bovid species, the *de novo* assemblies produced from trio-binning have dramatically improved the existing reference genome for domestic cattle. Highly continuous assemblies like these allow gene discovery within and interspecific comparisons between large and complex regions that are fragmented or lacking in both short-read and long-read diploid-derived genome assemblies (Hsieh et al. 2019; Vollger et al. 2019; Miga et al. 2020; Logsdon et al. 2020). These difficult to assemble regions are increasingly understood as playing important roles in disease biology, genome organization, gene regulation, and speciation. Here, we present 2 novel haploid *de novo* assemblies for 2 species of the Felidae, a domestic cat and an Asian leopard cat, generated by applying the trio-binning method to an F1 Bengal hybrid cat.

## Methods

### Biological Materials

The parent-offspring trio is composed of a random-bred domestic cat dam, an Asian leopard cat (*Prionailurus bengalensis euptilurus*) sire, and a female F1 Bengal cat offspring (“Amber,” aka LXD-97). Fibroblast cell lines were established for the F1 female and the Asian leopard cat sire (Pbe-53). DNA for the domestic cat (Fca-508) dam was extracted from white blood cells. The F1 hybrid was generated at the National Cancer Institute animal colony as part of the generation of an interspecies mapping panel (Menotti-Raymond et al. 1999, 2003; Davis et al. 2015).

### Nucleic Acid Library Preparation and Sequencing

#### Long-Read Library Preparation and Sequencing

High molecular weight genomic DNA was extracted using a modified salting-out protocol (Miller et al. 1988) followed by length quantification using the Pippin Pulse pulse-field gel system (Sage Science). DNA was quantified via Qubit fluorometric quantification (Thermo Fisher Scientific). PacBio SMRT libraries were size selected (~20-kb) on the Sage Blue Pippin and sequenced across 20 SMRT cells on the Sequel I instrument (V3 chemistry) to yield approximately 90x coverage.

#### Short-Read Library Preparation and Sequencing

Standard dual indexed Illumina fragment libraries (~300-bp average insert size) were prepared for the parent samples using the NEBNext Ultra II FS DNA Library Prep Kit (New England Biolabs Inc.). Libraries were assayed with fluorometric quantification using the Qubit (Thermo Fisher Scientific) and electrophoresis using the TapeStation (Agilent). Samples were sequenced to ~40x genome-wide depth of coverage with 2×150-bp reads using the NovaSeq 6000 Sequencing System (Illumina).

#### Hi-C Library Preparation and Sequencing

F1 Bengal fibroblasts from Amber were fixed as a monolayer using 1% formaldehyde for 10 min, divided into ~4.2 × 10^6^ cell aliquots, snap frozen in liquid nitrogen and stored at −80°C as described (Ramani et al. 2016). Cells were lysed, resuspended in 200ul of 0.5x DNase I digestion buffer, and chromatin digested with 1.5 units of DNase I for 4 min. Downstream library preparation was performed as described (Ramani et al. 2016) and sequenced across one Illumina HiSeq X Ten lane.

### Genome Assembly and Annotation

#### Haplotype Binning

A summary of software and versions used for each assembly step can be found in Table 1. All Illumina data was processed with *FastQC v0.11.8* (Andrews 2010) followed by adapter trimming using *Trim Galore! v0.6.4*. Parental Illumina sequences were used to phase the raw F1 Bengal PacBio long reads into domestic and Asian leopard cat haplotype bins using the trio binning feature of *Canu v1.8* (*TrioCanu*) (Koren et al. 2017; Koren et al. 2018). *TrioCanu* achieves this by identifying unique k-mers from the parental Illumina reads that are specific to each parental species. The greater the genetic divergence between the parents of the hybrid cross, the larger the number of species-specific k-mers that will be present within each PacBio long read to be classified as belonging to one parent or the other.

**Table 1.** Assembly pipeline and software usage

Assembly and Polishing	Software	Version
Haplotype Binning	Canu	v1.8
*De novo* Assembly	NextDenovo	v2.2-beta.0
Contig Polishing	NextPolish	v1.3.0
Benchmarking		
Basic Assembly Stats	QUAST	v5.0.2
Assembly Completeness	BUSCO	v4.0.6
Dotplot Generation	Nucmer	v4.0.0beta2
Dotplot Visualization	Dot	n/a
Scaffolding		
Hi-C Read Haplotyping	https://github.com/esrice/trio_binning	0.2.0
Hi-C Mapping for SALSA	https://github.com/esrice/slurm-hic/	n/a
Hi-C Scaffolding	SALSA2	v2.2
Ref-Based Scaffolding	RagTag	v1.0.1
Hi-C Contact Map Generation	Juicer	v1.5.7
Manual Assembly Inspection	Juicebox Assembly Tools	v1.11.08
Annotation		
Repeat Assessment	RepeatMasker	v4.0.9
Structural Variant Analysis	Assemblytics	v1.2.1
Annotation Liftover	Liftoff	v1.4.2

Software citations are listed in the text.

#### De novo Assembly

Haplotyped long reads for each species were assembled using *NextDenovo v2.2-beta.0* (NextDenovo 2020) with the configuration file (.cfg) altered for inputs: *minimap2_options_raw = -x ava-pb*, *minimap2_options_cns = -x ava-ont*. The *seed_cutoff=* option was adjusted to *8478* and *9777* for domestic and Asian leopard cat respectively.

#### Contig Polishing and QC

*NextPolish v1.3.0* (Hu et al. 2020) and *NextDenovo* corrected long reads were used to polish the raw contigs. Notable changes to the *NextPolish* configuration file included: *genome_size=auto*, and *task=best*, which instructs the program to perform 2 iterations of polishing using the corrected long reads. The *sgs* option was removed as polishing with the parental diploid short reads could lead to conversion of consensus sequence to reflect the alternate haplotypes not present in the F1. The *lgs* options within the configuration file was left at default settings except for modification for PacBio long reads by adjusting *minimap2_options= -x map-pb*. Basic assembly stats were generated using *QUAST v5.0.2* (Mikheenko et al. 2018) with the *--fast* run option selected. To assess genome completeness, *BUSCO v4.0.6* (Simão et al. 2015) was run using the *-m* genome setting with *-l mammalia_odb10* database selected (9226 single copy genes). Visual assessment of the haploid assemblies was performed through alignment to the felCat9 reference (GCA_000181335.4) (Buckley et al. 2020) using *nucmer* (*mummer3.23* package; Marçais et al. 2018) with default settings. The resulting delta file was used to generate a dot plot for genome comparison using *Dot: interactive dot plot viewer for genome-genome alignments* (Dot 2020).

#### Scaffolding

Polished contigs were scaffolded using Hi-C data generated from the F1 hybrid. Prior to scaffolding, F1 Bengal Hi-C reads were binned into parental haplotypes through alignment of the offspring reads to both polished parental assemblies using *bwa mem v0.7.17* (Li and Durbin 2009) and the *classify_by_alignment* (https://github.com/esrice/trio_binning/ v0.2.0) program as described in Rice et al. (2020). Haplotyped reads were mapped to polished contigs using the pipeline and scripts described in Rice et al. (2020) (https://github.com/esrice/slurm-hic/) using *SALSA v2.2* (Ghurye et al. 2017; Ghurye et al. 2019) with parameters *-e none -m yes.* The haplotyped Hi-C reads were used to scaffold each assembly followed by visual inspection of the SALSA scaffolds using *QUAST*, *nucmer*, and Hi-C contact maps. Following *SALSA*, *RagTag v1.0.1* (Alonge et al. 2019) was used to align scaffolds to their respective position in the felCat9 reference (Buckley et al. 2020) to identify any misassemblies. Selected *RagTag* parameters included *–remove-small, -f 10000* and *-j unplaced.txt*, a text file of scaffolds for *RagTag* to ignore based on their small size and identification as repetitive sequence in the *nucmer* alignments. *RagTag* scaffolds were manually inspected with Hi-C maps generated using *Juicer v1.5.7* (Durand et al. 2016a) with option *-s none* selected for compatibility with DNase Hi-C libraries. Maps were visualized using *Juicebox v1.11.08* (Durand et al. 2016b) and *Juicebox Assembly Tools* with scripts from *3d-dna v.180922* (Dudchenko et al. 2017).

#### Assembly Quality Control

Assembly quality control was performed by mapping Illumina short-read data from the biological parents, 3 unrelated domestic cats, and 3 unrelated Asian leopard cats to both reference assemblies (Supplementary Tables 1 and 2). Dictionaries were created for each reference fasta files using *Picard v2.21.6* CreateSequenceDictionary command. Short-read data was mapped using *bwa mem v0.7.17* (Li et al. 2009) and piped through *Samtools v1.3.1* (Li et al. 2009) view, sort, and index arguments. The sorted BAM files were processed in *GATK* with *v3.8.1* RealignerTargetCreator and IndelRealigner commands to fix indels. The realigned output sequences were then run through *ANGSD v0.925* (Korneliussen et al. 2014) to produce pseudo-haploid sequences from the diploid mappings and were subsequently split by chromosome using *pyfaidx v0.5.8* --split-files argument (Shirley et al. 2015). A multi-alignment file containing all mapped samples was created for each chromosome and parsed into 100kb windows using a custom script (see Data Availability). Pairwise uncorrected *p*-distance values were calculated per-window using a custom *p*-distance calculator script (see Data Availability). Assemblies were then evaluated through visual inspection of the *p*-distance traces across the reference genomes from both species to verify consistent separation of *p-*distances of the 2 species. Evidence of improper sorting would be indicated by a flip (high-to-low and low-to-high) in the *p*-distance signal of each respective species (all domestic cats and all Asian leopard cats, respectively).

#### Phased Haplotype Analysis

F1 interspecies hybrids are rare and, sometimes, acquisition of biological specimens from one or both biological parents may be difficult. Therefore, we sought to explore the prospects and limitations of using Illumina sequence data from non-biological parents with the long read phasing step in Trio-Canu. To evaluate how replacing one or both biological parents affected the haplotype sorting process, we developed a new script called *Phased Haplotype Analysis* (PHA) (see Data Availability). PHA takes the phased haplotype fasta files (maternal, paternal, and unknown) of a reference cross (biological x biological) and replacement cross (biological x non-biological or non-biological x non-biological) and compares the fasta files to identify correctly and incorrectly sorted reads. Correctly sorted reads are identified as reads that are phased to the same parental haplotype in both the reference and replacement crosses, whereas incorrectly sorted reads are identified as reads that were phased to a different parental haplotype in the replacement cross compared to the reference cross (Supplementary Figure 1). Reads identified as incorrectly sorted in the replacement cross are organized into different subtypes (i.e., maternal-to-paternal, maternal-to-unknown, etc.) (Supplementary Figure 1). PHA provides the number of reads correctly sorted into the same parental haplotype in both crosses, and provides a breakdown of the quantity of incorrectly sorted reads broken down into their respective subtypes. Further characterization of the incorrectly sorted reads was conducted with *RepeatMasker v4.0.7* (Smit et al. 2013–2015).

### Genome Annotation

#### Repeat Sequence Annotation

We used *RepeatMasker v4.0.9* (Smit et al. 2013–2015) with -*excln* and *-species cat* selected to identify and annotate repetitive regions of both genomes while ignoring gap sequence.

#### Structural Variant Analysis

To estimate indel rates and quantify repeat expansion and contractions we ran *Assemblytics v1.2.1* (web-based) (Nattestad and Schatz 2016) with a unique sequence length requirement of 10 000 on nucmer alignments between domestic and leopard cat single haplotype assemblies.

#### felCat9.0 Gene Annotation Liftover

Because of the high sequence similarity between the domestic and Asian leopard cat genomes, we used *Liftoff v1.4.2* (Shumate and Salzberg 2020) to perform an annotation liftover between the current felCat9 reference assembly (Buckley et al. 2020) and both *de novo* cat assemblies. Default parameters were used for all arguments except for calling *-copies* with *-sc 0.95* to identify extra copies of genes not previously annotated in felCat9.

## Results

### Sequencing and Assembly

**Figure 1.**
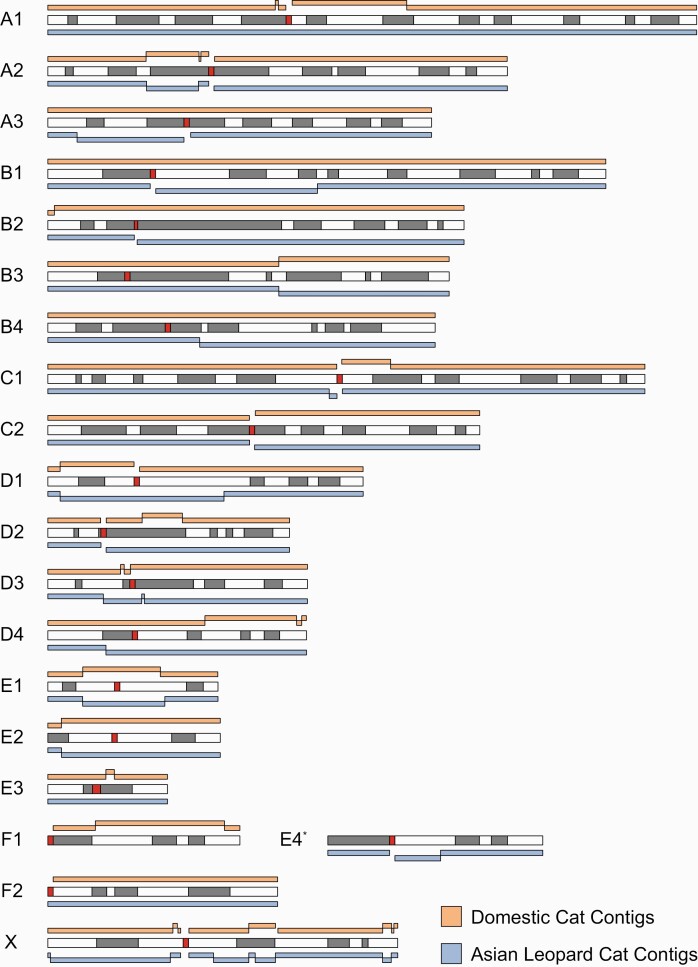
Alignment of domestic cat and Asian leopard cat single haplotype assembly contigs to felCat9. All ideograms are based on the domestic cat (Cho et al. 1997; Davis et al. 2009) except for the modified F1 to E4 chromosome unique to the species of the genera *Prionailurus*, *Acinonyx*, and *Puma* (Graphodatsky et al. 2020). G-banding is represented by dark bars and centromeres by red bars. Domestic cat contigs are depicted as orange bars above the ideogram, and Asian leopard cat contigs are depicted as blue bars below the ideogram.

All details pertaining to raw sequencing output are included in Supplementary Table 3. Genome assembly and sequencing metrics for the Domestic and Asian leopard cat haploid assemblies are found in Table 2. The number of haplotyped long reads from both parental species was very similar (Fca-508: 49.37%, Pbe-53: 50.62%, Unknown: 0.01%), as would be expected from an F1 individual. The number of assembled contigs for the domestic cat (*n* = 123) and Asian leopard (*n* = 132) cat were also similar. Contig N50 size was 83.88 Mb and 83.70 Mb for the domestic and leopard cat, respectively, a 100% increase relative to the diploid felCat9 long read assembly (contig N50 = 41.9 Mb) that was based on a highly inbred domestic cat of the Abyssinian breed. The largest contig was generated by the Asian leopard cat assembly, where chromosome A1, the largest cat chromosome, was captured in a single contig spanning the centromere (Figure 1). Contig alignments to felCat9 chromosomal sequences revealed that a majority of chromosome arms were captured in single contigs, and only 3 chimeric contigs were observed prior to scaffolding (1 in domestic cat, 2 in leopard cat) (Supplementary Figure 2). In the domestic cat assembly, 56% of autosomal chromosome arms were captured in single contigs and 85% in fewer than 2 contigs. The leopard cat assembly was similarly continuous, with autosomal chromosome arms being captured by 1 (60%) or fewer than 2 contigs (94%). Centromeres were captured within a single contig on 9 domestic cat and 10 leopard cat chromosomes. BUSCO analysis revealed that 95% of the 9226 mammalian BUSCOs were represented in each assembly with most (98%) being complete single-copy.

**Table 2.** Assembly statistics and benchmarks

Species	Domestic cat (2n = 38)	Asian leopard cat (2n = 38)
Read Count	6,342,174	6,519,732
Base Count (bp)	109,251,556,255	112,023,028,516
Subread N50 (bp)	25,541	25,585
Contig Assembly		
Total Contigs	123	132
Largest Contig (bp)	205,171,639	240,846,738
Ungapped Assembly Length (bp)	2,422,283,418	2,435,689,660
N50 (bp)	83,875,697	83,696,501
BUSCO (mammalia_odb10)		
Single-Copy	8,563	8,589
Duplicated	20	21
Complete	8,583	8,610
Percent Complete	93.03%	93.32%
Fragmented	166	153
Missing	477	463
Percent Present (Comp+Frag)	94.83%	94.98%
Scaffold Assembly Stats		
Total Scaffolds	71	83
Primary Assembly Length (bp)	2,422,299,418	2,435,702,060
Total Gaps	60	56
N50 Scaffold (bp)	147,603,332	148,587,958

Using a combined Hi-C and reference-based alignment approach we were able to obtain 19 chromosome length scaffolds that represented the conventional felid karyotypic arrangement of 18 autosomes and X chromosome. In the domestic cat assembly, 52 small scaffolds remained unplaced, representing just 0.41% of the un-gapped assembly length. The leopard cat contained 64 unplaced scaffolds composing 0.5% of the un-gapped sequence length. Scaffold alignments to felCat9 revealed the previously observed interchromosomal chimeric contigs were properly resolved (Supplementary Figure 3). Manual inspection using the Hi-C scaffolding data revealed no detectable misassemblies persisting for either assembly (Supplementary Figures 4 and 5). The total number of gaps introduced into each assembly was 60 (0.016 Mb) for the domestic cat and 56 (0.012 Mb) for the leopard cat (Supplementary Tables 4 and 5). The X chromosome, in particular, represented 28% (Fca) and 34% (Pbe) of all gaps, consistent with its enrichment for complex and ampliconic regions. The scaffold N50 of the final domestic and leopard cat assemblies were 147.60 and 148.59 Mb, respectively, approaching the theoretical maximum based on the domestic cat’s conventional chromosome lengths (Buckley et al. 2020). The leopard cat total genome length was 13.39 Mb longer than the domestic cat, which is likely due to variation in repetitive sequence amounts between the 2 species. This is supported by both RepeatMasker and Assemblytics structural variant analyses where we observed an 8.60 Mb increase in interspersed repeats and 9.19 Mb increase in gained sequence for the leopard cat when comparing the 2 assemblies (Supplementary Tables 6 and 7). Gene liftover from the felCat9 reference assembly to the single haplotype assemblies yielded a total of 19,569 and 19,457 protein-coding genes for domestic and leopard cat, respectively (Supplementary Table 8).

Genome alignments revealed 97.3% pairwise sequence identity between the domestic cat and Asian leopard cat chromosomes, estimated from 348,732 alignments of mean length=6.8-kb spanning 99% of the domestic cat assembly. The alignments also revealed no large structural rearrangements (Supplementary Figure 6). Two karyotypic differences were previously suggested to distinguish the 2 species: a pericentric inversion on Chr D2 and a putative pericentric inversion that distinguishes domestic cat Chr F1 (acrocentric) from Asian leopard cat Chr E4 (metacentric) (Wurster-Hill and Centerwall 1982). Genome alignments demonstrated that the D2 and F1/E4 homologs are grossly colinear between the 2 species and the latter difference in centromere location between F1 and E4 is the result of a *de novo* centromere repositioning event.

#### Assembly Quality Control

**Figure 2.**
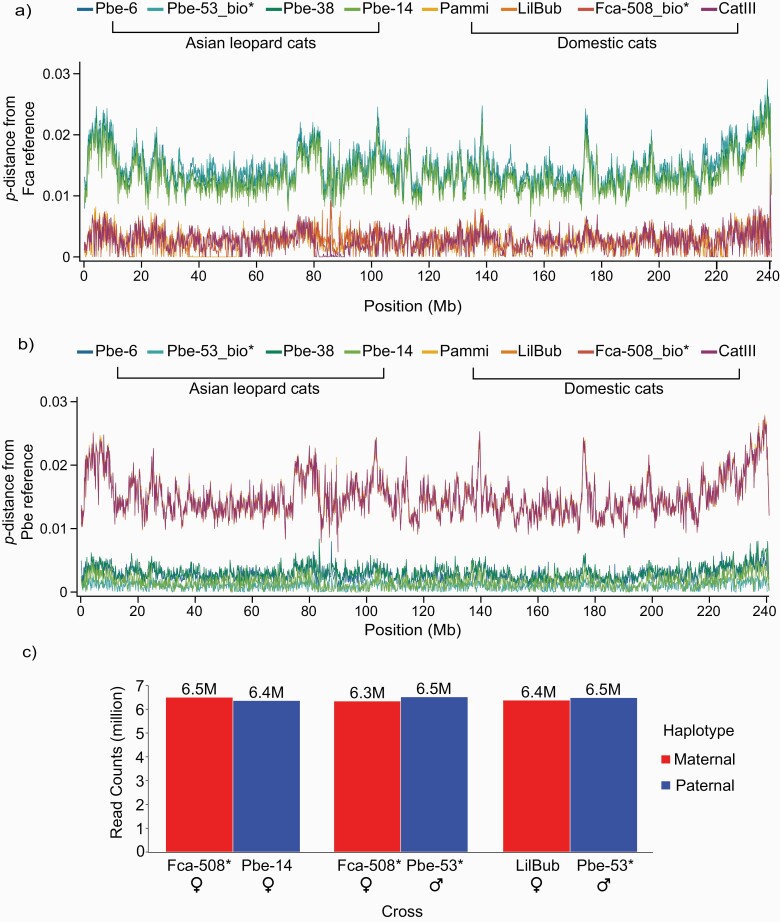
Read count distribution of single-replacement crosses and Chromosome A1 *p*-distance plots for both the domestic and Asian leopard cat reference sequences. (**a**) ***p***-distance traces for the biological parents and test sample short read data from both species mapped to the single haplotype domestic cat genome assembly. The Asian leopard cat samples show a clear separation from the traces of the domestic cat samples which lie close to 0. (**b**) *p*-distance traces for the biological parents and test sample short read data from both species mapped to the single haplotype Asian leopard cat genome assembly. In contrast to the *p*-distance traces for the domestic cat assembly, the Asian leopard cat traces lie close to 0, while the divergent domestic cat sample traces lie well above, indicating uniformly elevated divergence from the Asian leopard cat assembly. The consistent separation of reads from the 2 species in both (a) and (b) demonstrates that *TrioCanu* has properly phased the F1-hybrid long-read data into their appropriate parental haplotypes. (**c**) Read count distributions of single-replacement crosses post-haplotype phasing. (*) = Biological parents. See Supplementary Tables 1 and 2 for individual sample IDs.

To assess the phasing accuracy in our final haploid assemblies, we used *p*-distance, the proportion of nucleotide sites where 2 sequences differ, to determine if any reads/regions of the genome were improperly sorted during the initial haplotype phasing step of *TrioCanu*. For example, any region of the genome where the mapped domestic cat reads were more or equally similar to the reference Asian leopard cat genome than the other leopard cat sequences would be considered evidence of improperly phased sequences, or alternatively, past episodes of introgression (e.g., Rice et al. 2020). The full genome *p*-distance plots for both the Domestic cat (Supplementary Figure 7) and the Asian leopard cat (Supplementary Figure 8) show consistent separation of the domestic cat and Asian leopard cat *p*-distance traces across all chromosomes (Figure 2a,b). This indicates that *TrioCanu*’s haplotype phasing step properly binned the long-read data into their respective parental haplotypes.

We also evaluated *TrioCanu*’s ability to accurately phase the F1 hybrid long-read data by replacing the Illumina data from either the biological mother (Fca-508, domestic cat), the biological father (Pbe-53, Asian leopard cat), or both biological parents with data from other individuals. We used 3 unrelated domestic cats and 3 unrelated Asian leopard cat samples (Supplementary Table 1), one being the same subspecies (*P. bengalensis euptilurus*) as the Asian leopard cat sire, and 2 being from a different subspecies (*P. bengalensis bengalensis*). Our results produced nearly identical results from the haplotype sorting process with biological parents, with only a relatively small number of reads being phased to a different haplotype (Figure 2c). Further analysis revealed that the vast majority of incorrectly sorted reads (i.e., a read was sorted to a different haplotype in the replacement cross compared to the reference cross) were short in length (Supplementary Figure 9), with 79–80% of the reads being shorter than 10-kb in length (Supplementary Table9). We also analyzed the subtype distribution of the incorrectly sorted reads (i.e., incorrectly sorted from mother-to-father, father-to-mother, etc.) and found that the majority were switching between the maternal and paternal haplotypes (Supplementary Table 10), comprising just 3.5% (<3X mean coverage) of the total F1 hybrid sequence data. However, when we performed replacement crosses with Asian leopard cats from a divergent subspecies (*P. b. bengalensis*) or closely related species (*P. javanensis* or *P. viverrinus*) the phasing of the parental reads were increasingly skewed towards one parent (Supplementary Figure 10).

Finally, we reassembled the PacBio reads from LXD-97 after read phasing was performed with Illumina data from 2 different individuals (LilBub and Pbe-14) rather than the actual biological parents, Fca-508 and Pbe-53. The resulting domestic cat assembly aligned across 99.99% of the original Fca-508 assembly (Supplementary Figure 11a), and differed in assembly length by only 0.34%, with an average 99.98% sequence identity and an SNP rate of 0.001%. The Asian leopard cat assembly produced comparable results, aligning across 100% of the original Pbe-53 assembly (0.24% length difference) with 99.98% sequence identity and an SNP rate of 0.001% (Supplementary Figure 11b).

## Discussion

We have produced 2 highly continuous genome assemblies for the domestic cat (*Felis catus*) and the Asian leopard cat (*Prionailurus bengalensis*) by applying the trio-binning approach to long sequence reads from a Bengal F1 hybrid. Sequence continuity for these 2 assemblies is twice that of the most recent diploid-based long read domestic cat reference (Buckley et al. 2020) and is equivalent to that of the most recent haploid human genome assemblies (Miga et al. 2020). Sequence improvements and gains relative to the diploid felCat9 assembly include complex repetitive regions previously un-spanned due to insufficient read lengths and/or high haplotype divergence and resolution of multicopy gene families with high allelic diversity (i.e., Major Histocompatibility Locus, olfactory receptors). Furthermore, we have provided a genome assembly from a random-bred domestic cat, which is more representative of the domestic cat pet population.

In addition to improvements in the domestic cat reference genome, the simultaneous generation of a highly continuous Asian leopard cat genome will be a valuable resource for studying the population genetic diversity, subspecies delimitation and conservation with this species and other members of *Prionailurus*. This genome will also be valuable for health studies in closely related species, such as transition cell carcinoma in fishing cats and polycystic kidney disease in Pallas’ cats. High-resolution comparisons between a hybridizing pair of felid species will also be valuable for quantifying species-specific divergence across copy number variable regions, previously described in felids as being associated with hybrid sterility and speciation (Davis et al. 2015). The success of the trio-binning approach has stimulated the generation of other highly continuous genomes derived from additional felid F1 hybrids, like the Safari cat (domestic cat × Geoffroy’s cat) and liger (lion × tiger) (Bredemeyer et al. in prep.). Comparative genomic analyses from these high-quality assemblies will produce unprecedented insights into mechanisms underlying morphological divergence, adaptation, and speciation within this enigmatic mammalian family.

F1 interspecies hybrids are rare biological resources, and in many cases, it may be logistically impossible to obtain the actual parents of the cross. This motivated us to explore the feasibility of applying short-read data from other conspecifics to phase the F1 long-read sequences. We demonstrated that *TrioCanu*’s phasing is robust to the inclusion of Illumina short-read data from non-biological parents when one or both biological parents are missing, producing assemblies of virtually identical length and sequence identity with those produced from reads phased by the biological parents. However, under such circumstances, we recommend phasing with reads from an individual of the same subspecies or derived from a genetically similar population, as phasing errors increase with divergence from the parental species.

Felid genomes are known to be highly conserved across the family, with G-banding and FISH analyses showing gross co-linearity across the majority of feline autosomes and the X chromosome (Wurster-Hill and Centerwall et al. 1982; Modi and O’Brien 1988; Davis et al. 2009). Our study provides the first demonstration that the genomes of *Felis* and *Prionailurus*, although karyotypically distinct, are grossly colinear and that cytogenetic differences do not correspond to chromosomal rearrangements. This confirms recent genomic comparisons between the domestic cat and lion that also demonstrated gross collinearity across the deepest divergence of the cat family (Armstrong et al. 2020), and suggests an even more extreme level of karyotypic conservation within the Felidae than previously appreciated.

## Supplementary Material

esaa057_suppl_Supplementary_Material

## Data Availability

Raw sequence reads and assembly accessions are found under SRA BioProject PRJNA670214 (*Felis catus*) and PRJNA682572 (*Prionailurus bengalensis*). Scripts and other associated data for the PHA analysis can be found at the GitHub repository link https://github.com/eutherialab/Bengal_F1_Assembly.
