# Supplementary material for: Ultracontinuous Single Haplotype Genome Assemblies for the Domestic Cat (Felis catus) and Asian Leopard Cat (Prionailurus bengalensis)
Source: J Hered. 2020 Dec 11;112(2):165–73. doi: 10.1093/jhered/esaa057 (PMC8006817; doi:10.1093/jhered/esaa057)
Supplement: esaa057_suppl_Supplementary_Material [file esaa057_suppl_supplementary_material.pdf]

Supplementary Information for

Ultracontinuous single haplotype genome assemblies for the domestic cat and Asian leopard cat

Kevin R. Bredemeyer*, Andrew J. Harris*, Gang Li, Le Zhao, Nicole M. Foley, Melody Roelke-Parker, Stephen J. O'Brien, Leslie A. Lyons, Wesley C. Warren, and William J. Murphy

Supplementary Figure 1. Phased Haplotype Analysis Schema

Supplementary Figure 2. Contig alignments to felCat9

Supplementary Figure 3. Scaffold alignments to felCat9

Supplementary Figure 4. Domestic cat (Fca-508) Hi-C contact maps

Supplementary Figure 5. Asian leopard cat (Pbe-53) Hi-C contact maps

Supplementary Figure 6. Asian leopard cat assembly alignment to domestic cat assembly

Supplementary Figure 7. Full genome *p*-distance plots to the domestic cat reference

Supplementary Figure 8. Full genome *p*-distance plots to the Asian leopard cat reference

Supplementary Figure 9. Read length distribution graphs for incorrectly sorted reads in replacement crosses

Supplementary Figure 10. Single biological replacement read counts

Supplementary Figure 11. Nucmer alignments comparing assemblies generated from reads sorted using data from biological parents and non-biological parent trios.

Supplementary Table 1. SRA Identifiers

Supplementary Table 2. Sample information

Supplementary Table 3. Raw sequencing output and haplotyping results

Supplementary Table 4. Domestic cat chromosome assembly

Supplementary Table 5. Asian leopard cat chromosome assembly

Supplementary Table 6. RepeatMasker repeat analysis summary

Supplementary Table 7. Assemblytics structural variant analysis summary

Supplementary Table 8. Annotation liftover summary

Supplementary Table 9. PacBio read length distributions for six incorrectly sorted subtypes from two replacement crosses.

Supplementary Table 10. Percentage of incorrectly sorted reads by subtype

Supplementary Figure 1. Phase Haplotype Analysis read comparison for reference and replacement crosses. (a) Assuming *TrioCanu* sorted the reads correctly to the reference cross, green arrows indicate correctly sorted reads, while the dashed red lines indicate incorrect sorted reads in the replacement cross. (b) Organization of incorrectly sorted reads into subtypes that describe how the read was sorted differently compared to the reference cross.

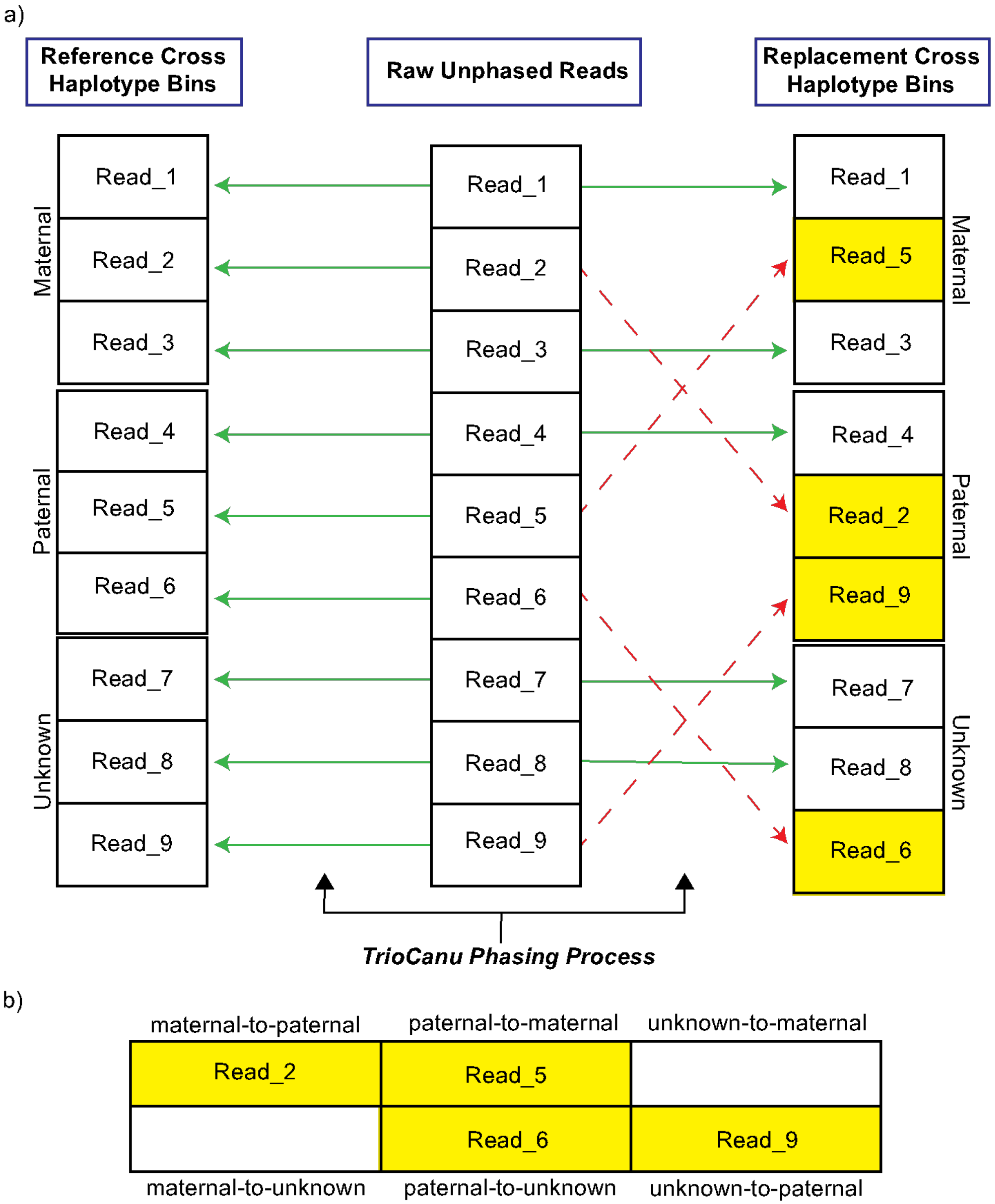

Supplementary Figure 2. Nucmer alignments of assembly contigs to the felCat9 reference genome identified chimeric contigs in the initial Nextdenovo assembly. **a)** Domestic cat polished contigs. Chimeric contig ctg000007 is an interchromosomal join between Chr D4 and Chr E2. **b)** Asian leopard cat polished contigs. Chimeric contig ctg000062 is an interchromosomal join between Chr B1 and Chr E4 (Chr F1 in felCat9) and chimeric contig ctg000126 is an interchromosomal join between Chr A2 and Chr E1.

a)

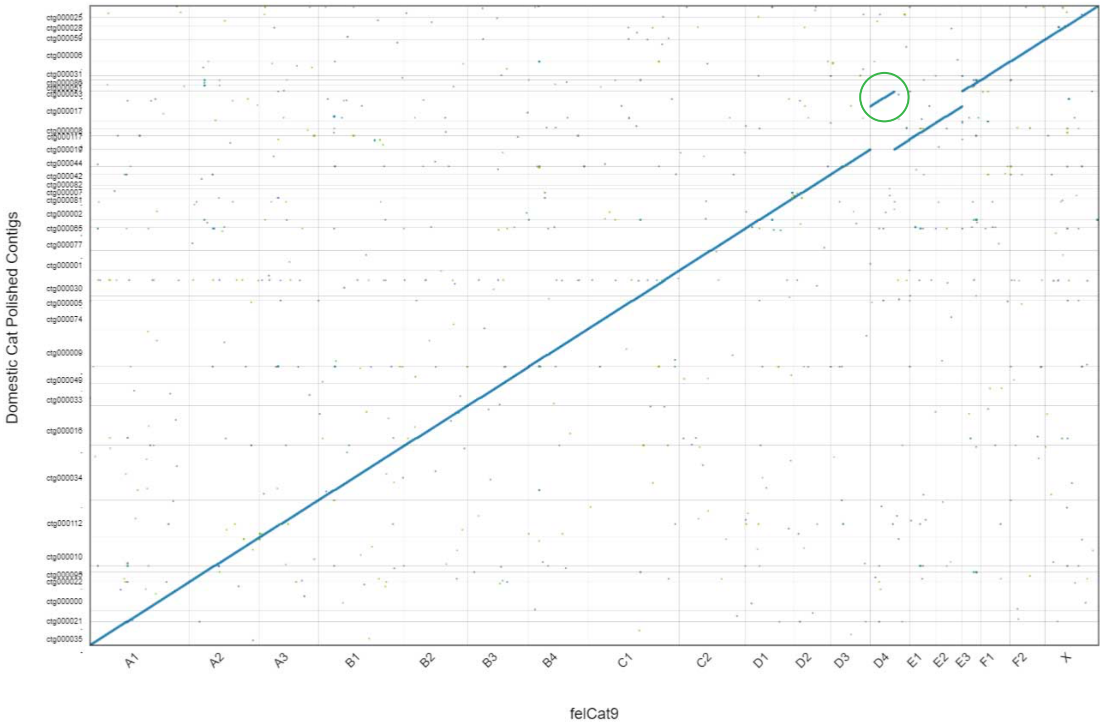

b)

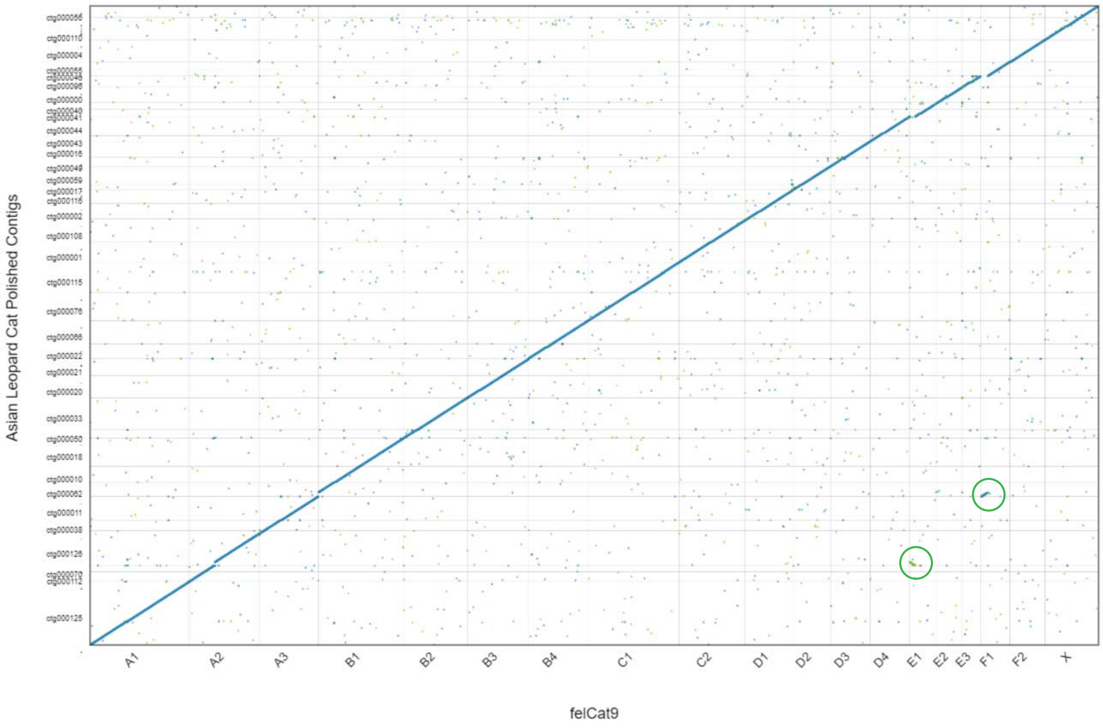

Supplementary Figure 3. Nucmer alignments of the final assembly scaffolds to the felCat9 reference genome. **a)** Domestic cat scaffolds. **b)** Asian leopard cat scaffolds.

a)

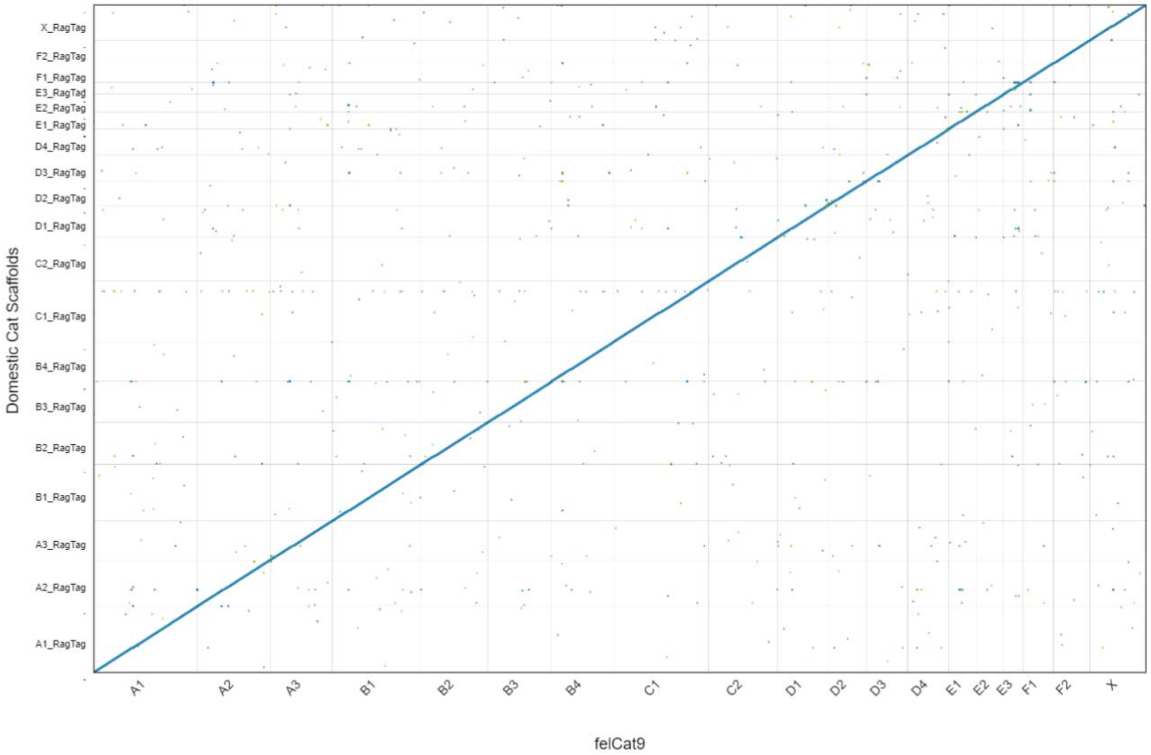

b)

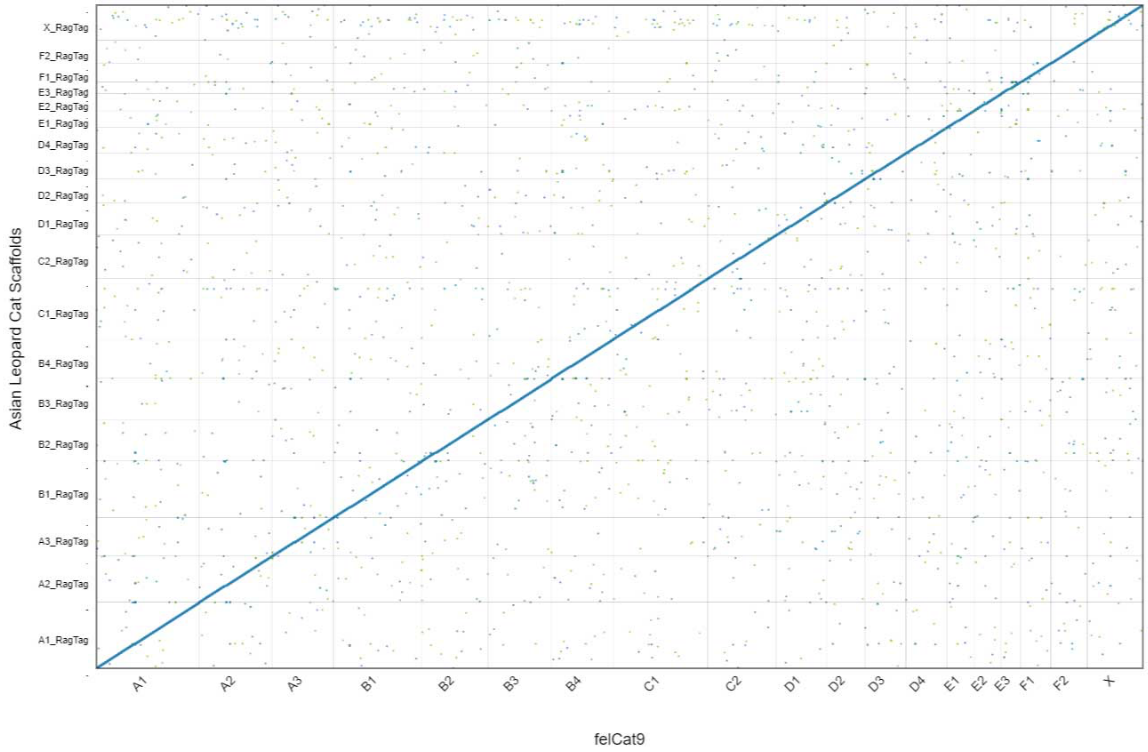

Supplementary Figure 4. Hi-C contact map for the domestic cat single haplotype assembly.

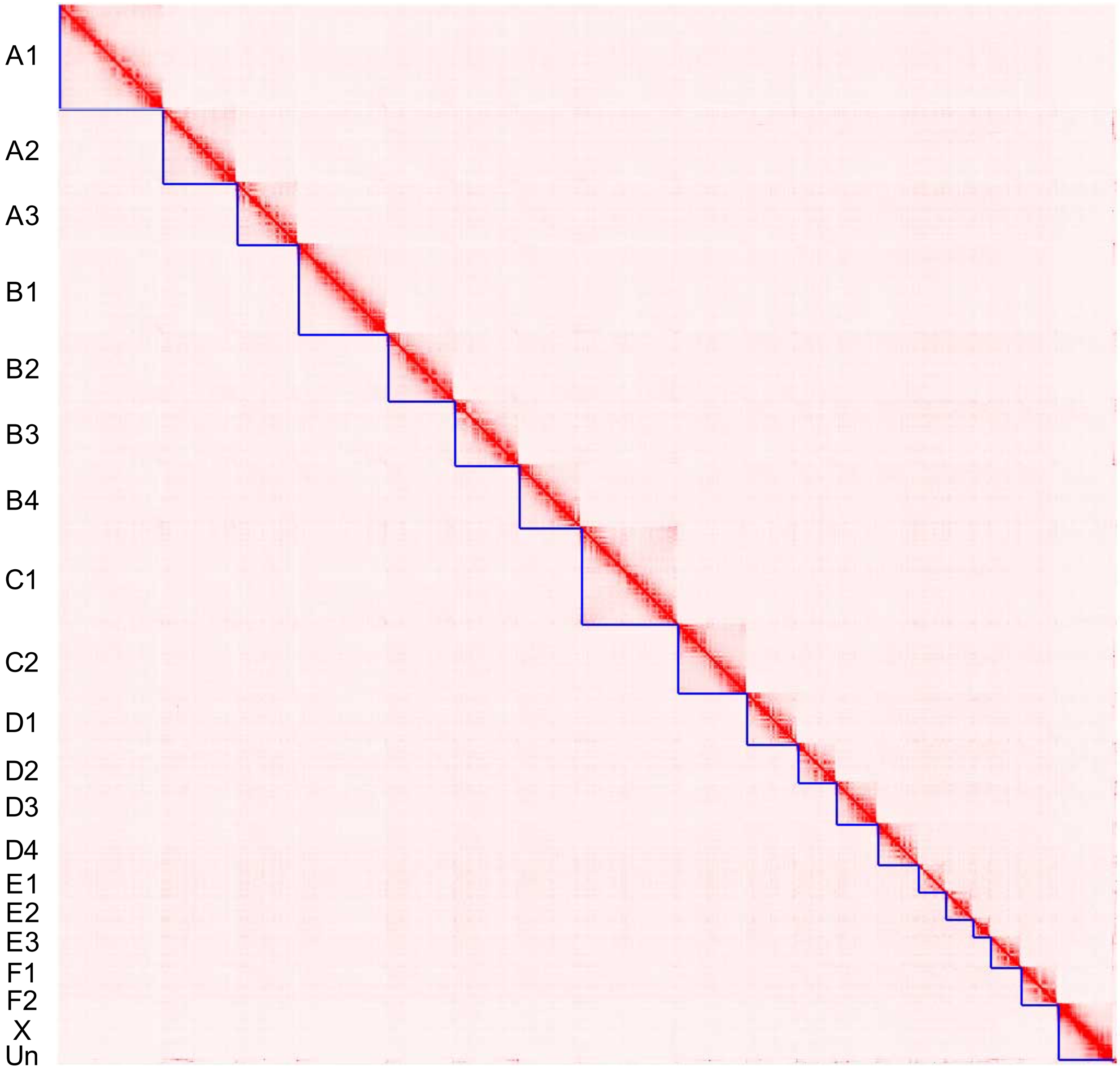

Supplementary Figure 5. Hi-C contact map for the Asian leopard cat single haplotype assembly.

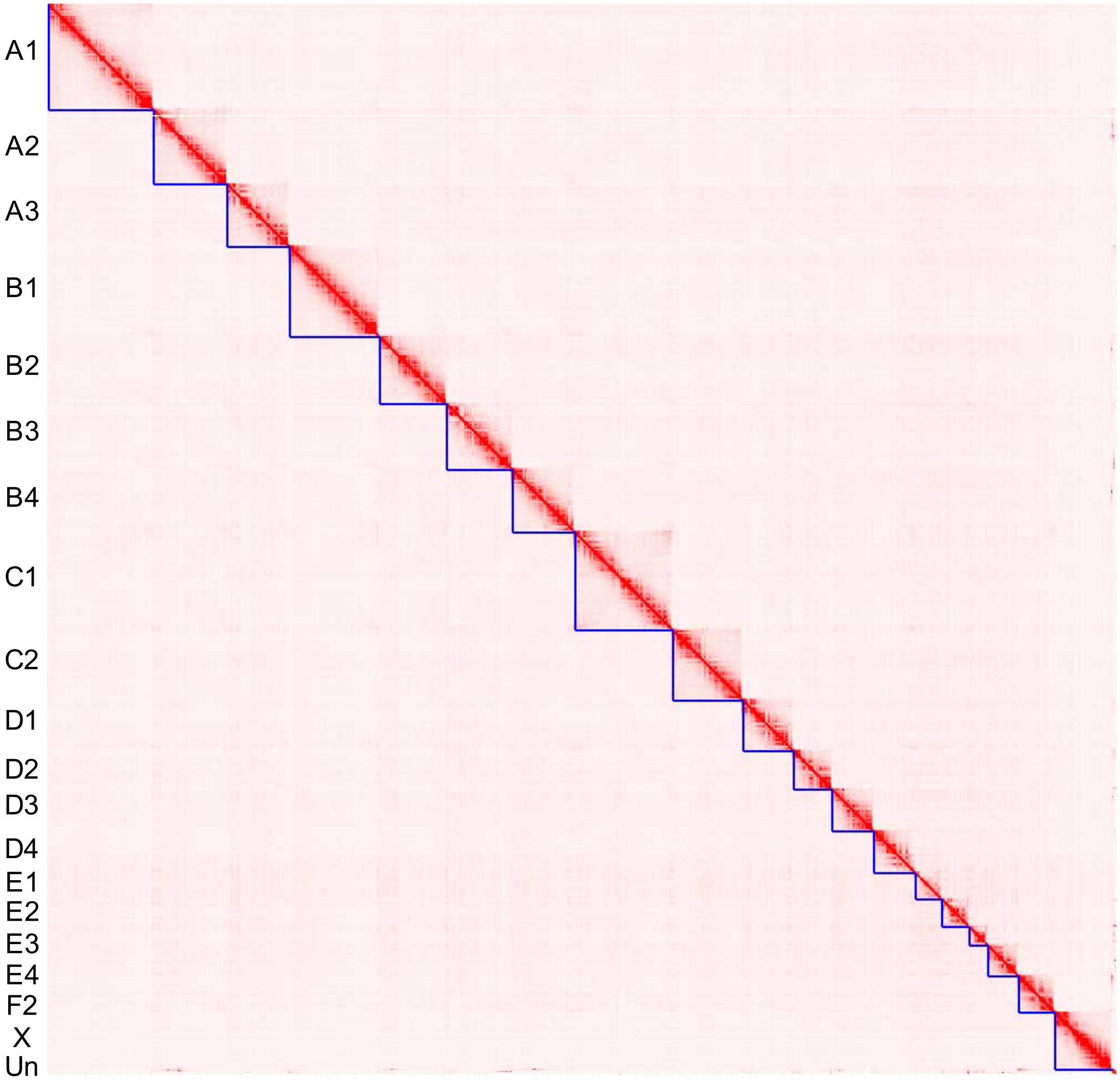

Supplementary Figure 6. Nucmer alignments of Asian leopard cat single haplotype assembly scaffolds to the domestic cat single haplotype assembly scaffolds.

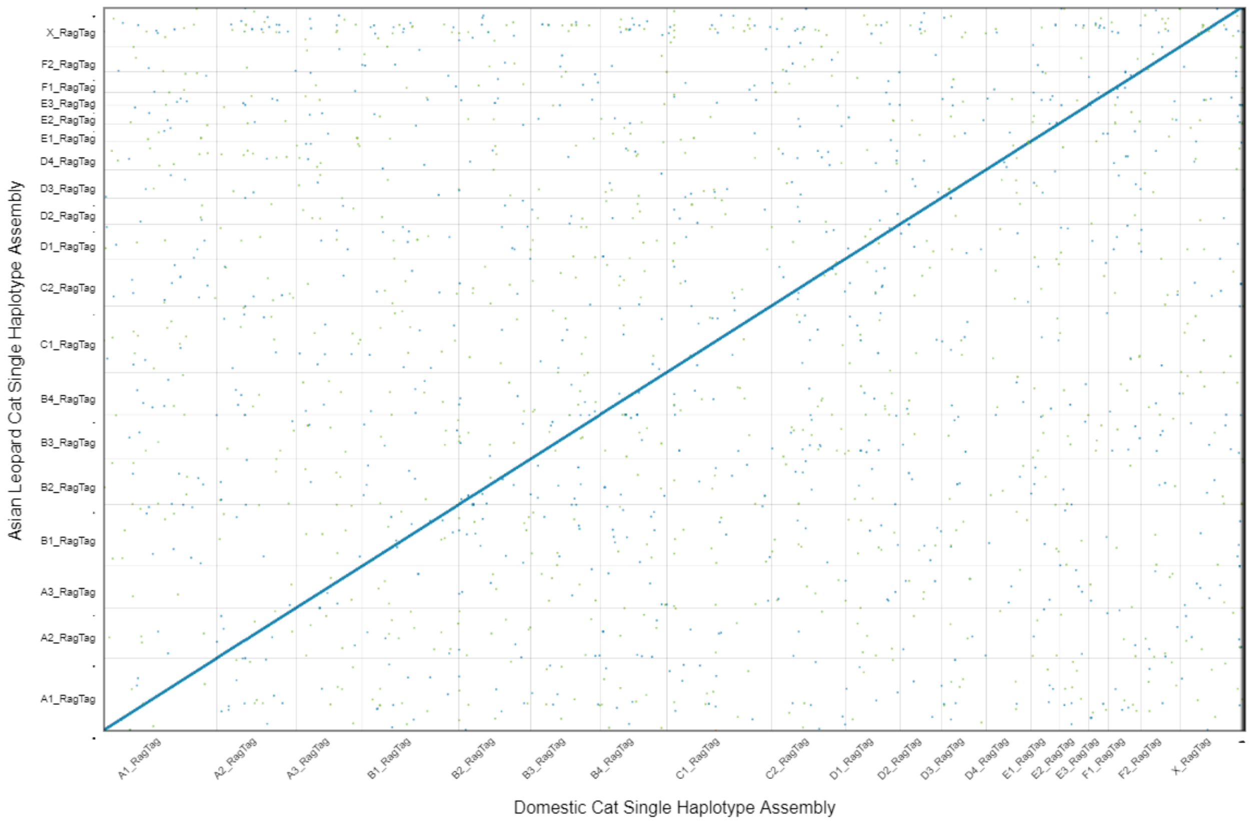

Supplementary Figure 7. *P*-distance traces of the 3 domestic cat (red/orange) and 3 Asian leopard cat (green/blue) test samples mapped to the Fca-508 single haplotype reference assembly. An interactive version of this plot can be found on GitHub.

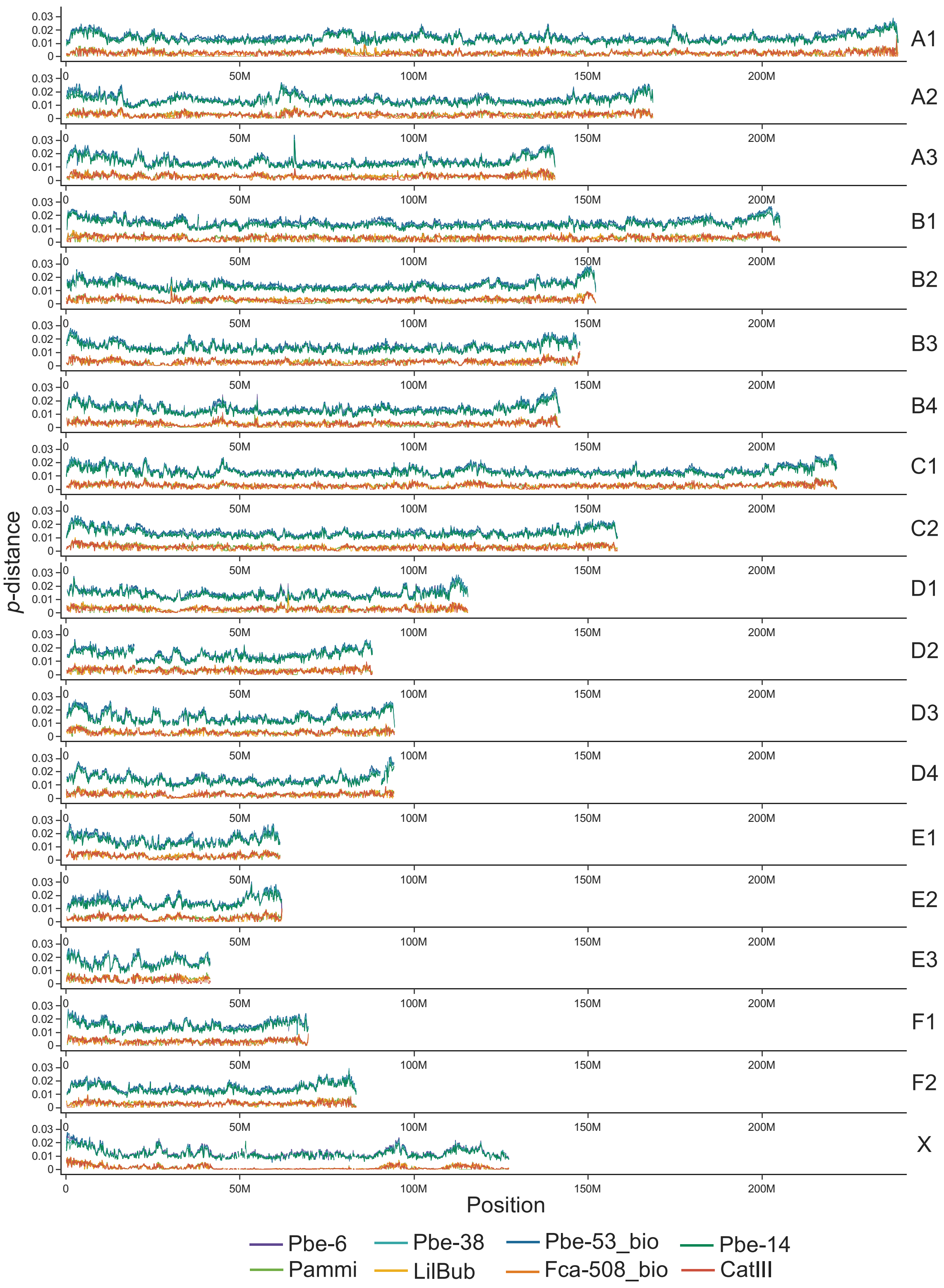

Supplementary Figure 8. *P*-distance traces of the 3 domestic cat (red/orange) and 3 Asian leopard cat (green/blue) test samples mapped to the Pbe-53 single haplotype reference assembly. An interactive version of this plot can be found on GitHub.

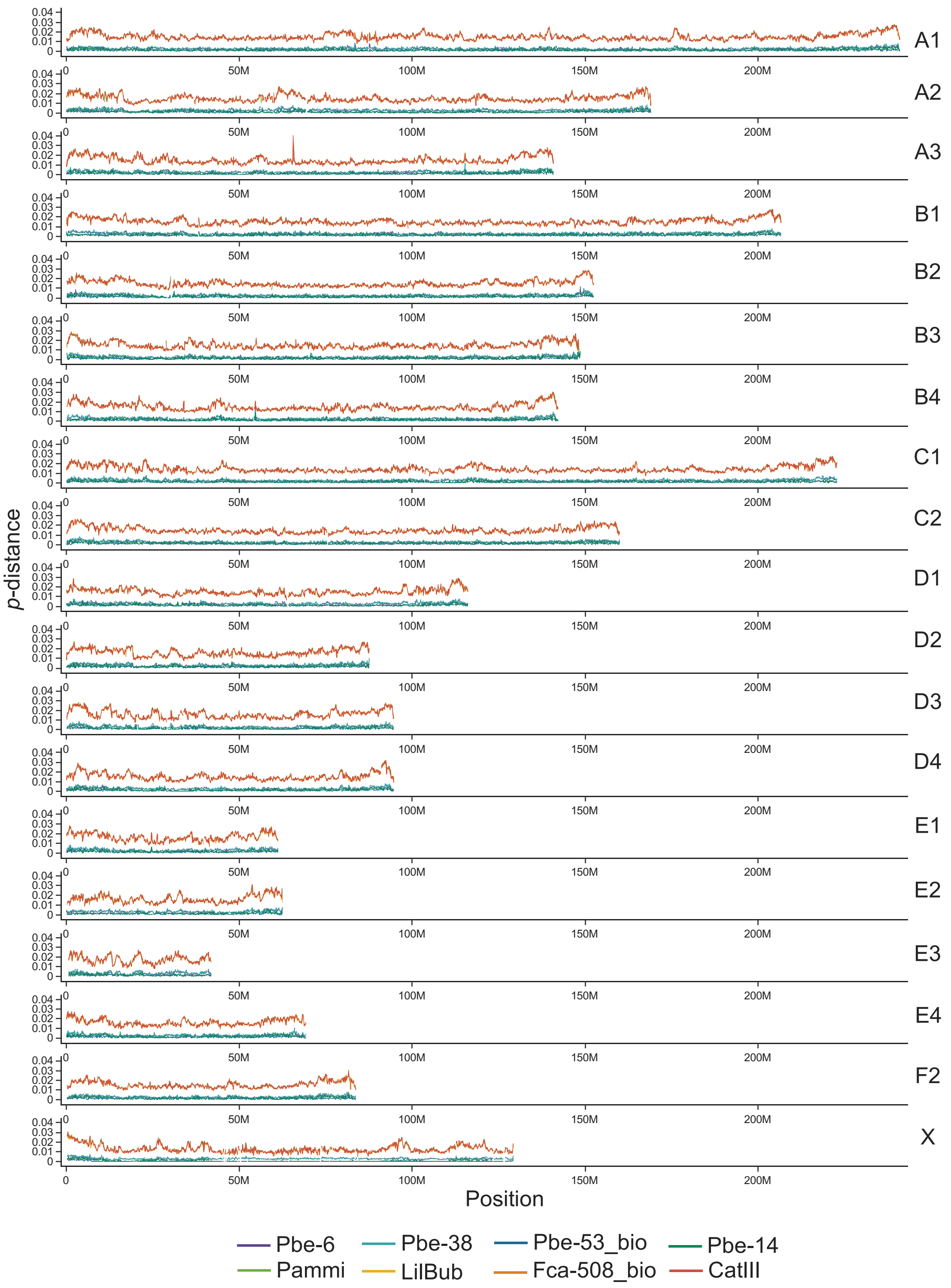

Supplementary Figure 9. PacBio read length distributions for six incorrectly sorted subtypes from two replacement crosses. 79.83% (LilBub x Pbe-53) and 80.29% (Fca508 x Pbe-14) of the incorrectly sorted reads are less than 10-kb in length, and 56.99% (LilBub x Pbe-53) and 51.83% (Fca508 x Pbe-14) of the incorrectly sorted reads are less than 5-kb in length. By comparison, the median read length for the correctly sorted reads were 14.7kb and 14.6kb for LilBub x Pbe-53 and Fca-508 x Pbe-14, respectively.

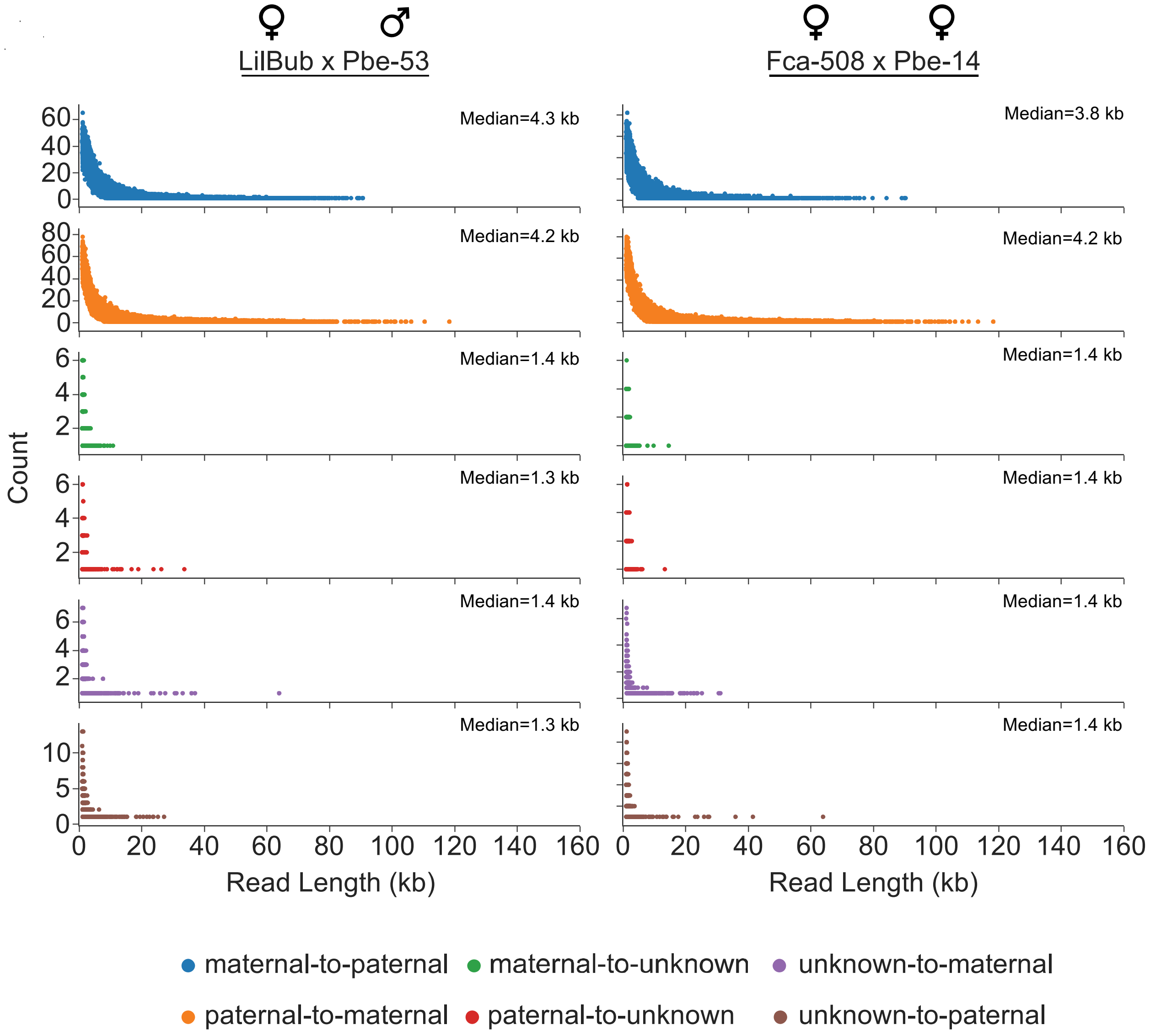

Supplementary Figure 10. Maternal and paternal haplotype read counts for single biological replacement crosses. We observe a tendency to skew read phasing toward one parental haplotype or the other when using increasingly divergent non-biological replacement parent samples (Pja-5, Pja-25, Pvi-12). (*) indicates biological samples, (E)= *Prionailurus bengalensis euptilurus*, (B)= *Prionailurus bengalensis bengalensis*, (Pvi)= *Prionailurus viverrinus*, (Pja)= *Prionailurus javanensis.*

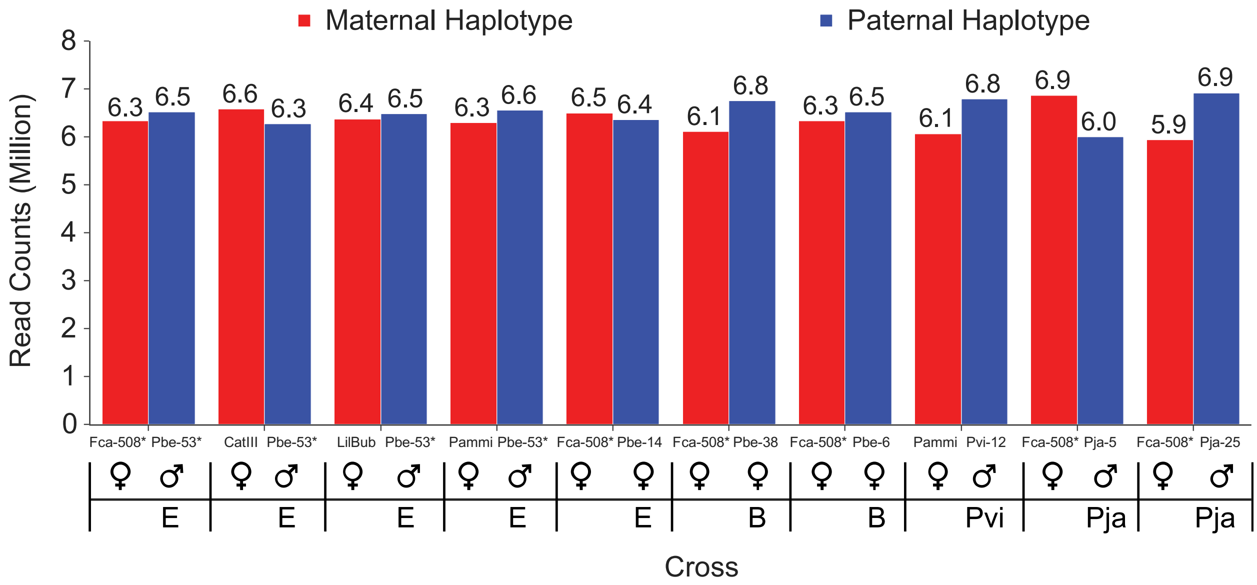

Sample	Species/Subspecies
Pja-25	*Prionailurus javanensis*
Pja-5	*Prionailurus javanensis*
Pvi-12	*Prionailurus viverrinus*
Pbe-14	*Prionailurus bengalensis euptilurus*
Pbe-38	*Prionailurus bengalensis bengalensis*
Pbe-6	*Prionailurus bengalensis bengalensis*

Supplementary Figure 11. Nucmer alignments comparing assemblies generated from reads phased using the biological parent short read data (Fca-508 x Pbe-53) and a replacement cross using two different parents (LilBub x Pbe-14). **a)** Alignment of domestic cat replacement cross contigs (*y* axis) to biological assembly (*x* axis). **b)** Alignment of Asian leopard replacement cross contigs (*y* axis) to biological assembly (*x* axis).

a)

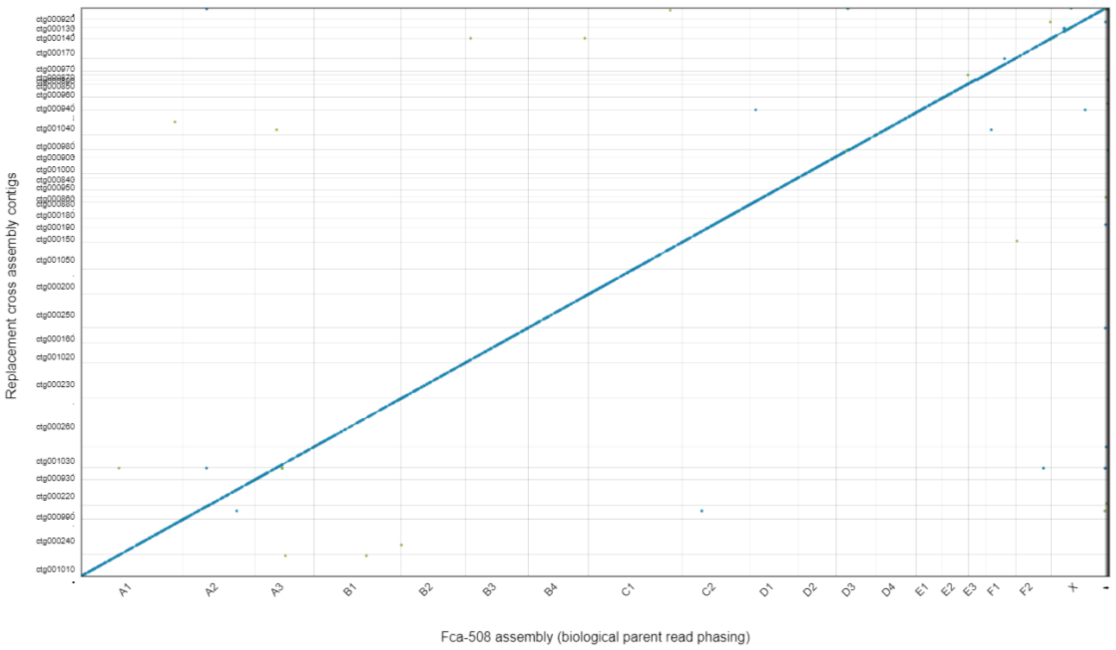

b)

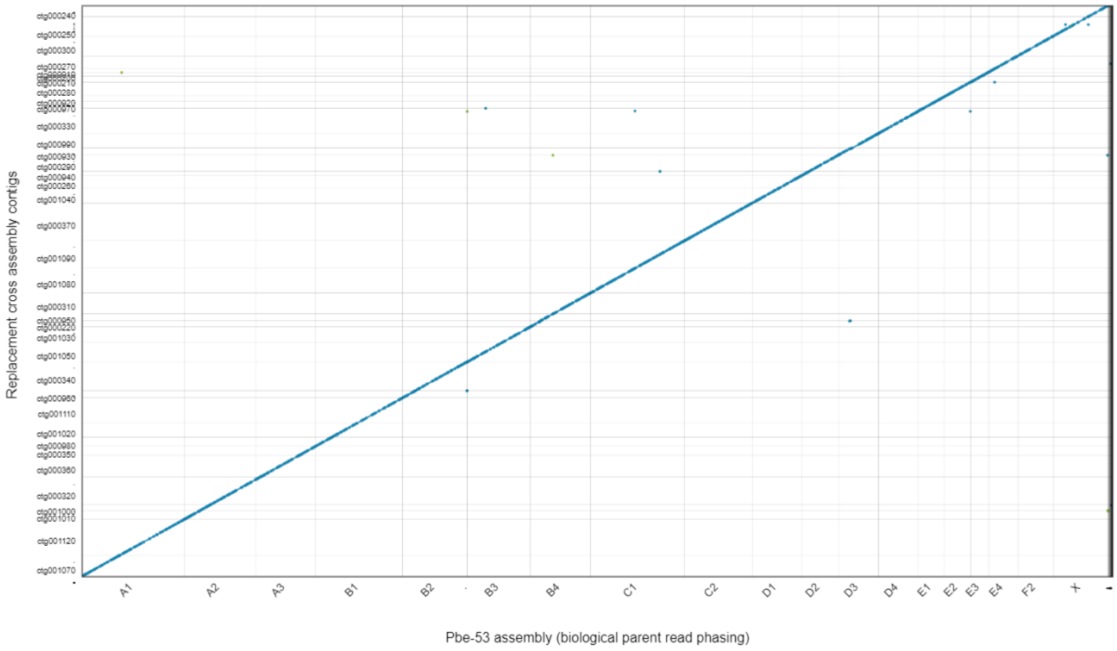

Supplementary Table 1. Sample accession information for additional Asian leopard cats (1-3), *Prionailurus* species (4-6), and domestic cats (7-9) used in the assembly QC and Phased haplotype analysis.

Sample ID	SRA ID	Species	SRA #	BioProject
1. Pbe-6	PBE	*Prionailurus b. bengalensis*	SRR2062628	PRJNA286910
2. Pbe-14	PBEP0014	*Prionailurus b. euptilurus*	SRR4426166	PRJNA348661
3. Pbe-38	PBEP0038	*Prionailurus b. bengalensis*	SRR4426179	PRJNA348661
4. Pja-5	PBEP0005	*Prionailurus javanensis*	SRR4426170	PRJNA348661
5. Pja-25	PBEP0025	*Prionailurus javanensis*	SRR4426162	PRJNA348661
6. Pvi-12	PVIP0012	*Prionailurus viverrinus*	SRR4426169	PRJNA348661
7. Pammi	100_Pammi	*Felis catus*	SRR8092623	PRJNA495843
8. CatIII	CatIII	*Felis catus*	SRR5815677	PRJNA393717
9. LilBub	LilBUB	*Felis catus*	SRR8377759	PRJNA512113

Supplementary Table 2. Sample and sequence information for the domestic cat, Asian leopard cat, and two other *Prionailurus* species used for assembly, assembly QC and the Phased Haplotype Analysis.

Library Name/ ID	Avg. Read Length	Base Count	Organism	Subspecies	Sex	Instrument
Pbe-53	150	52,451,028,300	*Prionailurus bengalensis*	*euptilurus*	M	Illumina NovaSeq 6000
Pbe-14	125	32,821,274,250	*Prionailurus bengalensis*	*euptilurus*	F	Illumina HiSeq 2500
Pbe-38	125	37,174,242,250	*Prionailurus bengalensis*	*bengalensis*	F	Illumina HiSeq 2500
Pbe-6	101	64,245,439,662	*Prionailurus bengalensis*	*bengalensis*	F	Illumina HiSeq 2000
Pja-5	125	31,492,238,250	*Prionailurus javanensis**	*sumatranus*	M	Illumina HiSeq 2500
Pja-25	101	40,850,158,010	*Prionailurus javanensis**	*sumatranus*	M	Illumina HiSeq 2000
Pvi-12	101	39,586,764,968	*Prionailurus viverrinus*	*viverrinus*	M	Illumina HiSeq 2000
Fca-508	150	71,002,000,000	*Felis catus*	N/A	F	Illumina NovaSeq 6000
Pammi	151	146,300,000,000	*Felis catus*	N/A	F	Illumina HiSeq X Ten
CatIII	137	82,600,000,000	*Felis catus*	N/A	F	Illumina HiSeq X Ten
LilBub	155	93,110,000,000	*Felis catus*	N/A	F	Illumina NextSeq 500

*These samples were reclassified as *Prionailurus javanensis sumatranus* after they were uploaded to NCBI

Supplementary Table 3. Raw sequencing output and haplotyping results.

PacBio Long Reads

Sample	Reads	Bases (bp)	Coverage	Subread N50 (bp)	Avg. Read Length (bp)
F1 Bengal	13,801,880	221,688,858,786	89x	25,561	16,062
Domestic Cat Haplotype	6,342,174	109,251,556,255	44x	25,541	17,226
Leopard Cat Haplotype	6,519,732	112,023,028,516	45x	25,585	17,182
Unknown Haplotype	11,876	20,811,380	.01x	1,667	1,752

Illumina Reads

Sample	Reads	Bases (bp)	Coverage
Domestic Cat Parent (Fca-508)	473,347,659	142,004,297,700	57x
Leopard Cat Parent (Pbe-53)	349,673,522	104,902,056,600	42x

***In situ* DNase Hi-C Reads**

Sample	Reads	Bases (bp)	Coverage*
F1 Bengal	481,008,096	144,302,428,800	58x
Domestic Cat Haplotype	273,559,977	82,067,993,100	33x
Leopard Cat Haplotype	278,398,530	83,519,559,000	33x

*The summed number of haplotyped reads is higher than the F1 raw total because reads mapping equally well to both assemblies were included in each haplotype (following Rice et al., 2020).

Supplementary Table 4. Domestic cat single haplotype chromosome assembly.

Molecule	Total Length (bp)	Gaps (#)	Un-gapped (bp)
**ALL**	**2,422,299,418**	**60**	**2,422,283,418**
Chromosome A1	239,109,665	4	239,108,865
Chromosome A2	168,571,291	6	168,569,491
Chromosome A3	140,469,438	0	140,469,438
Chromosome B1	205,171,639	0	205,171,639
Chromosome B2	152,154,423	2	152,153,823
Chromosome B3	147,603,332	1	147,603,232
Chromosome B4	141,964,754	0	141,964,754
Chromosome C1	221,453,569	2	221,453,369
Chromosome C2	158,479,582	1	158,479,482
Chromosome D1	115,437,799	2	115,437,599
Chromosome D2	88,171,565	3	88,170,865
Chromosome D3	94,347,243	3	94,346,543
Chromosome D4	94,319,450	5	94,317,350
Chromosome E1	61,581,190	2	61,580,590
Chromosome E2	61,931,696	1	61,931,596
Chromosome E3	41,490,710	2	41,490,510
Chromosome F1	69,616,193	2	69,615,193
Chromosome F2	83,330,349	1	83,330,249
Chromosome X	127,194,472	17	127,190,772
Unplaced (n=52)	9,901,058	6	9,898,058

Supplementary Table 5. Asian leopard cat single haplotype chromosome assembly.

Molecule	Total Length (bp)	Gaps (#)	Un-gapped (bp)
ALL	**2,435,702,060**	**56**	**2,435,689,660**
Chromosome A1	240,846,738	0	240,846,738
Chromosome A2	168,940,850	3	168,940,150
Chromosome A3	140,803,547	2	140,802,947
Chromosome B1	206,580,432	2	206,579,832
Chromosome B2	152,385,405	2	152,385,205
Chromosome B3	148,587,958	1	148,587,858
Chromosome B4	142,198,231	1	142,197,731
Chromosome C1	222,814,610	2	222,814,410
Chromosome C2	159,850,271	3	159,849,971
Chromosome D1	116,110,351	3	116,110,051
Chromosome D2	87,619,889	1	87,619,389
Chromosome D3	94,595,352	7	94,594,252
Chromosome D4	94,620,989	1	94,620,489
Chromosome E1	61,174,949	2	61,174,349
Chromosome E2	62,591,731	1	62,591,631
Chromosome E3	41,869,280	0	41,869,280
Chromosome E4	69,230,405	2	69,229,405
Chromosome F2	83,696,601	1	83,696,501
Chromosome X	129,104,405	19	129,100,905
Unplaced (n=64)	12,080,066	3	12,078,566

Supplementary Table 6. RepeatMasker repeat analysis summary.

	Domestic Cat Scaffolds			Leopard Cat Scaffolds		
# bp masked / % of total sequence	813,861,694		33.60%	822,029,473		33.75%
Elements	Number of Elements	Length Occupied (bp)	Percentage of Sequence	Number of Elements	Length Occupied (bp)	Percentage of Sequence
**SINEs**	**469,804**	**69,316,698**	**2.86%**	**468,697**	**69,148,368**	**2.84%**
Alu/B1	0	0	0.00%	0	0	0.00%
MIRs	462,205	68,412,320	2.82%	461,113	68,245,362	2.80%
**LINEs**	**818,687**	**458,240,914**	**18.92%**	**821,304**	**467,024,407**	**19.17%**
LINE1	454,137	361,491,179	14.92%	456,511	370,292,026	15.20%
LINE2	310,052	84,612,805	3.49%	310,346	84,619,403	3.47%
L3/CR1	40,752	8,854,769	0.37%	40,765	8,853,628	0.36%
RTE	12,505	3,083,991	0.13%	12,430	3,061,807	0.13%
**LTR elements**	**285,140**	**108,443,228**	**4.48%**	**284,988**	**108,405,945**	**4.45%**
ERVL	87,026	39,290,813	1.62%	87,082	39,352,413	1.62%
ERVL-MaLRs	145,738	50,919,236	2.10%	145,760	50,904,494	2.09%
ERV_classI	28,377	12,425,503	0.51%	28,350	12,375,675	0.51%
ERV_classIII	0	0	0.00%	0	0	0.00%
**DNA elements**	**342,481**	**68,413,225**	**2.82%**	**342,709**	**68,441,325**	**2.81%**
hAT-Charlie	193,904	36,333,016	1.50%	193,801	36,337,024	1.49%
TcMar-Trigger	53,529	14,398,204	0.59%	53,607	14,400,538	0.59%
Unclassified	3,746	584,939	0.02%	3,687	583,111	0.02%
**Total Interspersed Repeats**		**704,999,004**	**29.10%**		**713,603,156**	**29.30%**
small RNA	140,966	10,719,095	0.44%	140,848	10,718,354	0.44%
Simple Repeats	1,463,437	69,960,000	2.89%	1,463,778	68,700,051	2.82%
Low complexity	521,881	28,033,555	1.16%	538,647	28,857,794	1.18%

Supplementary Table 7. Assemblytics variant analysis comparing the leopard cat single haplotype assembly to the domestic cat single haploid assembly.

	Leopard Cat Scaffolds	
Variant	Count	Total (bp)
**Insertion**		
50-500	13,465	2,499,723
500-10,000	734	1,906,174
Total	14,199	4,405,897
**Deletion**		
50-500	13,288	2,189,922
500-10,000	564	1,090,944
Total	13,852	3,280,866
**Tandem Expansion**		
50-500	829	178,794
500-10,000	211	309,941
Total	1,040	488,735
**Tandem Contraction**		
50-500	611	136,116
500-10,000	78	82,503
Total	689	218,619
**Repeat Expansion**		
50-500	12,756	3,686,143
500-10,000	12,680	25,435,846
Total	25,436	29,121,989
**Repeat Contraction**		
50-500	11,482	3,187,343
500-10,000	10,340	18,142,475
Total	21,822	21,329,818

*Leopard cat gain/loss	9,187,318 bp
Total structural variants	77,038
Total affected bases	58.85 Mb

*Leopard cat gain/loss calculated by subtracting basepairs of sequence gained (insertions/expansions) from sequence lost (deletions/contractions).

Supplementary Table 8. Results of annotation liftover of protein-coding genes between the felCat9 reference and the new single haplotype assemblies reported in this paper.

	Protein Coding Genes		
	felCat9	Fca-508	Pbe-53
Chromosome A1	1,247	1,253	1,244
Chromosome A2	1,591	1,600	1,599
Chromosome A3	1,131	1,135	1,133
Chromosome B1	966	974	969
Chromosome B2	1,077	1,069	1,000
Chromosome B3	1,187	1,204	1,194
Chromosome B4	1,225	1,232	1,220
Chromosome C1	1,737	1,760	1,742
Chromosome C2	898	905	904
Chromosome D1	1,448	1,467	1,466
Chromosome D2	631	640	632
Chromosome D3	661	668	666
Chromosome D4	798	812	809
Chromosome E1	1,096	1,099	1,099
Chromosome E2	1,107	1,108	1,106
Chromosome E3	691	692	689
Chromosome F1/E4	681	690	685
Chromosome F2	422	424	422
Chromosome X	781	799	796
ChrUn	219	38	82
**Total**	**19,594**	**19,569**	**19,457**
**Difference**	**0.00%**	**0.13%**	**0.70%**

Supplementary Table 9. Incorrectly sorted read sequence content from the PHA analysis.

Cross	Total reads sorted	# incorrectly sorted reads	<50% repetitive	% reads <10kb	% reads <5kb
CatIII x Pbe-38	12,873,782	523,782	87.39%	79.06%	57.25%
Fca-508 x Pbe-14	12,873,782	362,052	88.53%	80.29%	58.31%
LilBub x Pbe-53	12,873,782	429,613	87.65%	79.83%	56.99%
LilBub x Pbe-6	12,873,782	519,606	90.95%	79.70%	57.93%

Supplementary Table 10. Percentage of incorrectly sorted reads separated by subtype. (M) = Maternal haplotype, (P)=Paternal haplotype, (U)=Unknown haplotype.

Cross	Mean Read Length (bp)	M-to-P	P-to-M	U-to-M	U-to-P	M-to-U	P-to-U
LilBubxPbe-53	6,992	44.85 %	53.38%	0.47%	0.75%	0.32%	0.23%
Fca-508xPbe-14	6,961	27.40%	70.65%	1.12%	0.54%	0.12%	0.17%
LilBubxPbe-6	7,206	40.42%	57.55%	0.67%	0.55%	0.37%	0.44%
CatIIIxPbe-38	7,365	47.17%	50.84%	0.65%	0.59%	0.36 %	0.39%
